# A case of pulmonary fibrosis associated with rheumatoid arthritis, scleroderma sine scleroderma and ANCA associated vasculitis

**DOI:** 10.1186/2193-1801-3-513

**Published:** 2014-09-10

**Authors:** Amritpal Singh Anand, Priya Brian Joseph, Ernest Vera-Vazquez

**Affiliations:** Lakeland Rheumatology, 3950 Hollywood Road, 280, St Joseph, MI 49085 USA; Lakeland Regional Medical Center, 1234 Napier Ave, St Joseph, MI 49085 USA

**Keywords:** Diffuse parenchymal lung diseases, Pulmonary fibrosis, Rheumatoid arthritis, Scleroderma, ANCA associated vasculitis

## Abstract

**Introduction:**

Diffuse parenchymal lung disease (DPLD) may be idiopathic or may be due to known associations such as autoimmune diseases. The prognosis in cases associated with autoimmune diseases depends on many factors such as histopathology, baseline lung function, auto-antibody level, et cetera. DPLD and its prognosis is worse in patients with overlap syndromes.

**Case description:**

We present a rare case of a 71 year old Caucasian lady with gradually worsening pulmonary fibrosis secondary to an overlap syndrome with rheumatoid arthritis (RA), scleroderma sine scleroderma (SSS) and anti neutrophil cytoplasmic antibody (ANCA) associated vasculitis.

**Discussion and Evaluation:**

In this paper, we share information from review of literature regarding DPLD associated with RA, systemic sclerosis (SSc) and ANCA associated vasculitis. Details of our discussion include prognostic factors, histology and radiographic features of these individual disease entities.

**Conclusion:**

Since pulmonary fibrosis in overlap syndromes has a poor prognosis, extensive work up should be performed even when clinical evidence of only one autoimmune disease is present.

## Introduction

Diffuse parenchymal lung disease (DPLD) is a heterogeneous group of disorders characterized by inflammation and/or fibrosis of the parenchymal interstitium of the lung. Progression of disease results in impaired oxygen transfer and scarring within the lungs (Morgenthau and Padilla 2009). It can be idiopathic or associated with other diseases. Many autoimmune diseases such as rheumatoid arthritis (RA), systemic sclerosis (SSc), dermatomyosits/polymyositis and anti neutrophil cytoplasmic antibody (ANCA) associated vasculitis can result in DPLD.

DPLD associated with autoimmune diseases can have either usual interstitial pneumonia (UIP) or non-specific interstitial pneumonia (NSIP) pattern in histology. UIP pattern has worse prognosis compared to NSIP. Also, UIP and NSIP have characteristic pattern in high resolution computed tomography (HRCT) scan of chest. UIP is characterized by basal dominance, peripheral reticular abnormalities, traction bronchiectasis and honeycombing. Honeycombing, while part of the classic appearance of UIP, is absent initially. The major HRCT feature in NSIP is ground glass appearance. However ground glass opacities may be present as the first manifestation of UIP or during acute exacerbations (Morgenthau and Padilla 2009).

The prognosis depends on many factors like histopathology, baseline lung function, auto-antibody level, et cetera. Although rare, there can be an overlap between various autoimmune conditions, each of them independently contributing to pulmonary fibrosis (PF). For example, ANCA positivity is associated with increased incidence of PF in RA patients (Cambridge et al. 1994). Similarly, increased incidence of PF is seen in SSc patients with ANCA positivity (Derrett-Smith et al. 2013) as well as in SSc-RA overlap patients (Szücs et al. 2007). As expected, the prognosis of PF in overlap syndromes is worse than individual entities (Cambridge et al. 1994; Derrett-Smith et al. 2013; Szücs et al. 2007). Also, ANCA positivity in PF can predispose to development of ANCA associated vasculitis (AAV) (Arulkumaran et al. 2011). Here, we present a case of PF with RA, scleroderma sine scleroderma (SSS) and AAV which has never been reported before and has a grave prognosis.

## Case report

A 71 year old Caucasian female, with history of chronic cough and dyspnea on supplemental oxygen was referred to our clinic with complaints of pain in the wrists, proximal interphalangial (PIP) joints and metacarpophalangial (MCP) joints of hands bilaterally. Associated symptoms included morning stiffness lasting longer than one hour as well as dysphagia. The cough and dyspnea had gradual onset and had been progressively worsening over the past few years. There was no associated fever, chills, sputum production, lower extremity edema, unintentional weight changes, orthopnea or exposure to inhaled occupational irritants. Her past medical history was significant for gastroesophageal reflux disease (GERD) and hypertension for which she was on Omeprazole and Lisinopril respectively. Lisinopril was discontinued and replaced with Amlodipine due to increased cough. However, she reported no change in her symptoms. Family history of RA (brother, maternal aunt) and lung cancer (father) was noted. She is a former smoker who quit smoking 37 years ago. At that time, she used to smoke 0.5 pack per day for 14 years. Initial investigations did not reveal any significant abnormality other than mildly elevated erythrocytic sedimentation rate (ESR) of 26 and C-reactive protein (CRP) of 0.9.

On physical examination, coarse crepitations were heard bilaterally in both lung fields, worse at the base. She could make only 50% of fist on right and 25% of fist on left and had a weak hand grip. Squeeze test was positive at the MCP and metatarsophalangial (MTP) joints. The PIP joints of hands and MCP joints were tender, warm and swollen. Elbow extension was decreased by 20 degrees on the left and by 5 degrees on the right. Squatting was incomplete due to bilateral knee discomfort. Skin examination noted several telangiectasias on her face. Nail fold capillaroscopic examination showed no significant abnormality.

**Figure 1:**
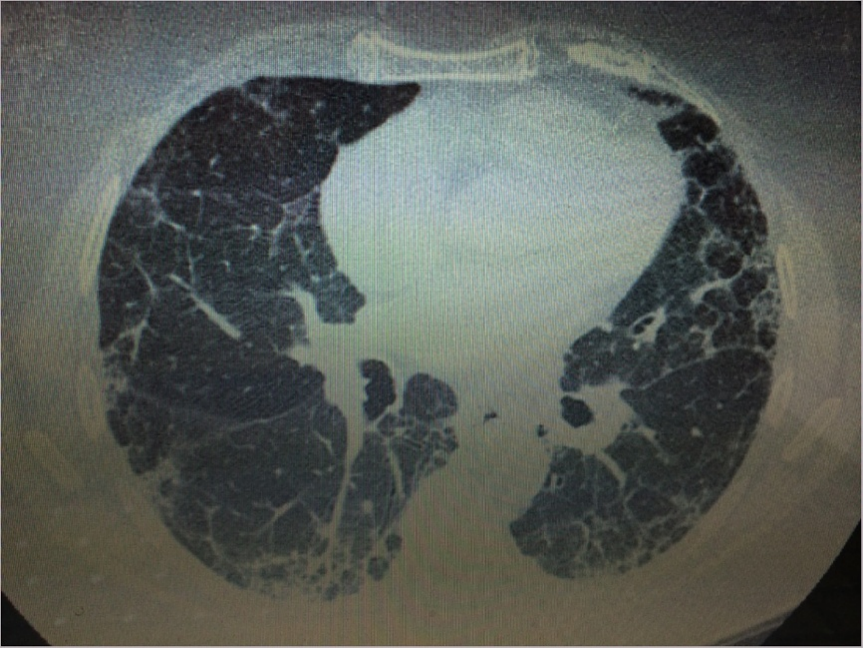
**HRCT scan demonstrating PF with UIP.**

Results of laboratory work up are detailed in Table 1. X-ray of feet showed erosive changes at the PIP, MTP and distal interphalangial (DIP) joints. Periarticular osteopenia and narrowing of the joint space were also noted at multiple joints in both hands and feet. HRCT chest showed widespread peripheral interstitial fibrotic change and mild honeycombing consistent with UIP (Figure 1 HRCT scan demonstrating PF with UIP).

**Table 1:** **Laboratory data**

Variable	Normal result	Patient result
WBC count, cells/mm^3^	3,600-12,700	8300
Abolute Neutrophil count, cells/mm^3^	1,800-7,700	5,200
Absolute Lymphocyte count, cells/mm^3^	1,000-4,800	3,700
Absolute Monocyte count, cells/mm^3^	100-1,000	700
Absolute Eosinophil count, cells/mm^3^	0-400	300
Absolute Basophil count, cells/mm^3^	0-500	100
Hemoglobin, gm/dl	12.0-16.0	13.7
Hematocrti,%	35.0-47.0	40.6
Platelet count, cells/mm^3^	140,000-440,000	280,000
ESR, mm/hour	0-30	*63*
CRP, mg/dl	<0.5	*0.9*
International normalized ratio	<1.0	1.1
Partial thromboplastin time, seconds	25-36	31.8
Glucose, mg/dl	65-100	92
Sodium, mmloes/litre	136-143	141
Potassium, mmloes/litre	3.6-5.0	4.3
Chloride, mmloes/litre	96-107	102
Bicarbonate, mmloes/litre	22-31	26
Urea nitrogen, mg/dl	6-30	12
Creatinine, mg/dl	0.5-1.0	0.7
Creatine kinase(CK), units/litre	30-135	74
Alanine transaminase, units/litre	9-52	30
Aspartate transaminase, units/litre	5-40	23
Urine analysis		Normal
Rheumatoid factor (RF), units/ml	<12	*111*
Anti-cyclic citrullinated protein (CCP) antibody, units/ml	<3.0	*6954.1*
Anti- nuclear antibody (ANA)	<1:40	*1:640 with centromeric pattern*
Anti-centromere antibody, AI	<1.0	*>8.0*
Anti-myeloperoxidase (MPO) antibody, AI	<1.0	*>8.0*
Anti- proteinase 3 (PR3) antibody,AI	<1.0	<0.2
Anti-ds-DNA, units /ml	<5.0	1.0
Anti- Smith antibody, AI	<1.0	<0.2
Anti- ribonucleoprotein (RNP) antibody, AI	<1.0	0.7
Anti- SSa antibody, AI	<1.0	0.3
Anti- SSb antibody, AI	<1.0	<0.2
Anti- Scl-70 antibody,AI	<1.0	<0.2
Anti- RNA polymerase 3 antibody, units	<19	14
Anti-Jo-1 antibody, AI	<1.0	<0.2
C3 complement, mg/dl	88-165	123
C4 complement, mg/dl	14-44	15
Beta-2 Glyco 1 IgG, units/ml	<7.0	2.0
Beta-2 Glyco 1 IgM, units/ml	<7.0	16
Anticardiolipin IgG, units/ml	<10.0	1.7
Anticardiolipin IgM, units/ml	<10.0	26
Lupus Anticoagulant	Negative	Negative

Pulmonary function test (PFT) was done which revealed restrictive pattern with forced expiratory volume in 1 second (FEV1) of 1.72 (89% of the predicted) and a reduced diffusion capacity for Carbon monoxide (corrected DLCO) of 62%. Transthoracic echocardiogram (TTE) demonstrated normal cardiac structure, function and ejection fraction except for pulmonary artery pressure of 37 mm of Hg. Open lung biopsy could not be performed due to high surgical risk of pulmonary complications. Biopsy specimen from lungs obtained through bronchoscopy was suggestive of extensive interstitial fibrosis with honey combing.

Patient was started on Hydroxychloroquine, Rituximab and low dose Prednisone at 10 mg per day. Higher doses of Prednisone could not be started due to potential risk of scleroderma renal crisis. Leflunomide and Minocycline were later added to the treatment regimen to manage RA. Overtime, Prednisone was tapered off as joint symptoms improved along with down trending inflammatory parameters. Seven months later, she developed nasal septal perforation along with worsening of inflammatory markers. Prednisone and Trimethoprim-Sulfamethoxazole were started. Otorhinolaryngologist advised against surgical correction and recommended conservative management. Subsequently in time, nasal perforation healed without any complications.

Currently, her treatment regimen consists of low dose Prednisone (10 mg per day), Hydroxychloroquine, Rituximab, Leflunomide and Minocycline with which her joint symptoms are under good control. She is still dyspneic and remains on supplemental oxygen without any clinical worsening.

## Discussion

PF is a well known complication in a number of autoimmune diseases (Morgenthau and Padilla 2009). The prognosis in these patients depends on several factors like histology, baseline lung function, presence of auto-antibodies and its titer level (Morgenthau and Padilla 2009). This is worse in overlap syndromes (Cambridge et al. 1994; Derrett-Smith et al. 2013; Szücs et al. 2007).

In our patient, there was evidence of more than one autoimmune condition. The joint symptoms, seropositivity, presence of anti-cyclic citrullinated protein (CCP) antibodies as well as X-ray evidence of erosions in the joints pointed towards aggressive form of RA. Factors which justified the diagnosis of SSS were positive ANA with centromeric pattern with high titer anti-centromere antibody, telangiectasia and GERD without obvious cutaneous involvement. In addition, myeloperoxidase (MPO)-ANCA positivity manifesting as nasal septal perforation and worsening of the inflammatory markers led to the diagnosis of AAV. Since PF in our patient appears to be associated with three different autoimmune processes, her prognosis is poor.

In order to recognize all the underlying processes contributing to PF, patients in whom suspicion of autoimmune disease is high, need to be worked up extensively. This evaluation should include complete blood count (CBC), comprehensive metabolic panel (CMP), ESR, CRP, creatine kinase, urine analysis, rheumatoid factor (RF), anti-CCP antibody, anti-nuclear antibody (ANA) by immuno fluorescence assay, anti-ds-DNA antibody, anti-Smith antibody, anti- ribonucleoprotein (RNP) antibody, anti-SSa & SSb antibody, anti-Scl-70 antibody, anti-centromere antibody, anti-RNA polymerase 3 antibody, anti-Jo-1 antibody, ANCA by immunofluorescence, anti-MPO antibody, anti-proteinase 3 (PR3) antibody, PFT, HRCT chest, EKG, TTE, bronchoalveolar lavage (BAL) and open lung biopsy if feasible. In the discussion that follows, we share information regarding DPLD associated with RA, SSc and AAV. Details of our discussion will include prognostic factors, histology and HRCT features of these individual disease entities.

### DPLD in RA

Most lung disease occurs within 5 years after the initial diagnosis of RA (Brown 2007). Clinically significant RA-DPLD occurs in nearly 10% of the RA population (Olson et al. 2011). Interstitial Lung Disease (ILD) is the only complication of RA reported to be increasing in prevalence (Bongartz et al. 2010). In a study by Kelly et al, 47% of deaths in patients with RA associated ILD (RA-ILD) were directly due to ILD (Kelly et al. 2014).

The factors found to be associated with the higher risk for development of ILD in RA were male gender, age, presence of inflammatory arthritis, disease activity, history of smoking, high titer RF and anti-CCP antibodies (Bongartz et al. 2010; Turesson et al. 2003).

The prognosis of RA-ILD has been reported to be poor in previous studies with mean survival of just 2.6 years from diagnosis (Bongartz et al. 2010). As a group, they have a better prognosis than those with idiopathic pulmonary fibrosis (IPF). Histopathology in these patients can be either UIP or NSIP, with UIP being more common. RA-ILD patients, who have UIP, have a survival similar to that of matched subjects with IPF (Park et al. 2007; Kim et al. 2010).

Data from idiopathic interstitial pneumonia (IIP) population show that HRCT chest can accurately predict the presence of histopathologic UIP pattern in a subset of patients (Hunninghake et al. 2003) and several studies in RA-ILD have suggested similar specificity (Assayag et al. 2014). So UIP pattern on HRCT is clinically relevant and all RA-ILD patients should undergo evaluation of their HRCT pattern. HRCT is widely available, reliable in hands of experienced radiologists, low cost and low risk compared to surgical lung biopsy. Patients with UIP pattern and extensive fibrosis on HRCT should be counseled on their poor prognosis and appropriate patients should be considered for lung transplantation (Kim et al. 2010).

### Systemic sclerosis and DPLD

The prevalence of ILD in patients with SSc varies from 25-90% depending on the clinical subtypes of SSc and the methods selected to define ILD (White 2003). ILD is the leading cause of morbidity and mortality in SSc (Rubio-Rivas et al. 2014). It is found more frequently in diffuse cutaneous scleroderma (72.7%) than limited scleroderma (16%) (Ostojić and Damjanov 2006). Also, it is reported in patients without any cutaneous sclerosis (scleroderma sine scleroderma) (Fischer et al. 2006). The extent and severity of cutaneous involvement does not correlate with pulmonary involvement (Morelli et al. 1997).

Although a variety of histopathologic patterns of ILD can occur in SSc, study by Fischer et al showed, NSIP to be the most common histopathologic pattern and with much better prognosis than UIP pattern. Median survival time for those with NSIP was 15.3 years compared with 3 years for those with UIP (Fischer et al. 2008). The HRCT features in patients with SSc are very similar to that of patients with idiopathic NSIP and are characterized by ground glass opacification (Desai et al. 2004). Another noteworthy point from the study by Fischer et al is that most of the patients did not have obvious scleroderma (skin involvement and positivity for specific antibodies). In addition, patients who presented with advanced disease had a poor outcome than those with early presentation. This heightens the need for earlier recognition and intervention (Fischer et al. 2008).

### Pulmonary fibrosis and ANCA positivity

The occurrence of AAV and ILD in the same patient, though rare is being increasingly recognized. Patients with initial diagnosis of PF, occasionally acquire seropositivity for MPO-ANCA, which may develop to microscopic polyangitis (MPA) (Ando et al. 2013). The prevalence of ANCA in patients with ILD in literature is suggested at 8-36% (Nozu et al. 2009; Foulon et al. 2008). Homma et al showed positive ANCA as an unfavorable prognostic factor in patients with pulmonary fibrosis (Homma et al. 2004). Nozu et al demonstrated that a high titer of ANCA is associated with a poor prognosis and higher risk for progression to MPA (Nozu et al. 2009). In fact, in a study by Foulan et al, 41% of patients with PF and ANCA positivity developed MPA over time (Foulon et al. 2008).

The study by Ando et al showed HRCT pattern of sub pleural reticular opacities, traction bronchiectasis and honeycombing in MPO-ANCA positive PF. Histologic features of MPO-ANCA positive PF were compatible with UIP pattern in which alveolar hemorrhage and small vessel vasculitis was not observed. The presence of pulmonary eosinophilia and low attenuation areas on CT scans might be predictive of MPO-ANCA positive conversion. Ando et al suggests measurement of MPO-ANCA during follow up periods if there is presence of eosinophilia in BAL and low attenuation areas on CT scans initially (Ando et al. 2013).

The presence of MPO-ANCA has been postulated to have a pathological role in the development of PF. Subclinical alveolar hemorrhage is often detected in patients with ANCA-associated vasculitis and this may contribute to pulmonary fibrosis (Birnbaum et al. 2007). MPO-ANCA antibodies have also been suggested to cause direct tissue injury by release of products of activated neutrophils with subsequent fibrosis (Birnbaum et al. 2007; Foucher et al. 1999). Alternatively, given that in majority of cases, IPF precedes development of MPA, the possibility of IPF inducing MPO-ANCA antibodies and MPA cannot be discounted (Foulon et al. 2008).

In addition, tobacco smoke exposure may play a part in initiating the pathophysiology of the disease. Tobacco smoke may irritate the epithelial cells, resulting in MPO expression. Also, tobacco smoke can induce a neutrophilic infiltration of the lung structures. This may contribute to self-immunization to myeloperoxidase or other neutrophil-associated proteins and the generation of ANCA with subsequent vasculitis. (Foulon et al. 2008).

Serial measurement of MPO-ANCA is recommended in patients with PF as ANCA positivity suggests increased risk of vasculitis and also to monitor disease activity once AAV develops. Early intervention may improve outcome in these patients as reducing ANCA titer with corticosteroid therapy may prevent development of MPA (Ando et al. 2013).

### Overlap syndromes

Presence of more than one autoimmune condition, contributing to PF has been reported and studied previously. For example, ANCA positivity appears to be associated with an increased incidence of lung fibrosis in RA patients (Cambridge et al. 1994). Positive ANCA test associated with anti MPO or anti PR3 antibodies with clinical evidence of vasculitis is rare in SSc patients. However, lung fibrosis occurs in around 80% of the SSc/AAV overlap patients far more frequently than would be expected for either disease individually. This susceptibility to lung fibrosis may be related to an inflammatory process or oxidative stress by ANCA in patients primed to develop fibrosis because of their SSc (Derrett-Smith et al. 2013). Similarly, study by Szucus et al in SSc-RA overlap patients showed pulmonary fibrosis in 77% of patients which is much higher than individual diseases alone (Szücs et al. 2007).

## Conclusion

In patients with PF and high suspicion of autoimmune disease, extensive work up should be undertaken as overlap syndrome may be a reasonable possibility. Needless to say, we recommend evaluating for additional immune mediated causes of PF even if one etiology is apparent as it can significantly influence prognostic outcome. It will be interesting to see in future, whether the overlap syndromes are true separate disease entities. The cases associated with more than one autoimmune conditions need to be reported, as such cases are not extensively studied. As noted in this case, HRCT may be as an alternative for lung biopsy especially if surgery is not feasible, as there is a good correlation between histopathology and HRCT findings. It is essential to note that these patients, if ANCA positive may develop AAV later in disease course. In time, if these individual disease entities are monitored and better controlled, then these patients may have better outcomes.

The manuscript does not contain clinical case studies or identifiable patient data.

## References

[CR1] Ando M, Miyazaki E, Ishii T, Mukai Y, Yamasue M, Fujisaki H, Ito T, Nureki S, Kumamoto T (2013). Incidence of myeloperoxidase anti-neutrophil cytoplasmic antibody positivity and microscopic polyangitis in the course of idiopathic pulmonary fibrosis. Respir Med.

[CR2] Arulkumaran N, Periselneris N, Gaskin G, Strickland N, Ind PW, Pusey CD, Salama AD (2011). Interstitial lung disease and ANCA-associated vasculitis: a retrospective observational cohort study. Rheumatology (Oxford).

[CR3] Assayag D, Elicker BM, Urbania TH, Colby TV, Kang BH, Ryu JH, King TE, Collard HR, Kim DS, Lee JS (2014). Rheumatoid arthritis-associated interstitial lung disease: radiologic identification of usual interstitial pneumonia pattern. Radiology.

[CR4] Birnbaum J, Danoff S, Askin FB, Stone JH (2007). Microscopic polyangiitis presenting as a “pulmonary-muscle” syndrome: is subclinical alveolar hemorrhage the mechanism of pulmonary fibrosis?. Arthritis Rheum.

[CR5] Bongartz T, Nannini C, Medina-Velasquez YF, Achenbach SJ, Crowson CS, Ryu JH, Vassallo R, Gabriel SE, Matteson EL (2010). Incidence and mortality of interstitial lung disease in rheumatoid arthritis: a population-based study. Arthritis Rheum.

[CR6] Brown KK (2007). Rheumatoid lung disease. Proc Am Thorac Soc.

[CR7] Cambridge G, Williams M, Leaker B, Corbett M, Smith CR (1994). Anti-myeloperoxidase antibodies in patients with rheumatoid arthritis: prevalence, clinical correlates, and IgG subclass. Ann Rheum Dis.

[CR8] Derrett-Smith EC, Nihtyanova SI, Harvey J, Salama AD, Denton CP (2013). Revisiting ANCA-associated vasculitis in systemic sclerosis: clinical, serological and immunogenetic factors. Rheumatology (Oxford).

[CR9] Desai SR, Veeraraghavan S, Hansell DM, Nikolakopolou A, Goh NS, Nicholson AG, Colby TV, Denton CP, Black CM, du Bois RM, Wells AU (2004). CT features of lung disease in patients with systemic sclerosis: comparison with idiopathic pulmonary fibrosis and nonspecific interstitial pneumonia. Radiology.

[CR10] Fischer A, Meehan RT, Feghali-Bostwick CA, West SG, Brown KK (2006). Unique characteristics of systemic sclerosis sine scleroderma-associated interstitial lung disease. Chest.

[CR11] Fischer A, Swigris JJ, Groshong SD, Cool CD, Sahin H, Lynch DA, Curran-Everett D, Gillis JZ, Meehan RT, Brown KK (2008). Clinically significant interstitial lung disease in limited scleroderma: histopathology, clinical features, and survival. Chest.

[CR12] Foucher P, Heeringa P, Petersen AH, Huitema MG, Brouwer E, Tervaert JW, Prop J, Camus P, Weening JJ, Kallenberg CG (1999). Antimyeloperoxidase-associated lung disease. An experimental model. Am J Respir Crit Care Med.

[CR13] Foulon G, Delaval P, Valeyre D, Wallaert B, Debray MP, Brauner M, Nicaise P, Cadranel J, Cottin V, Tazi A, Aubier M, Crestani B (2008). ANCA-associated lung fibrosis: analysis of 17 patients. Respir Med.

[CR14] Homma S, Matsushita H, Nakata K (2004). Pulmonary fibrosis in myeloperoxidase antineutrophil cytoplasmic antibody-associated vasculitides. Respirology.

[CR15] Hunninghake GW, Lynch DA, Galvin JR, Gross BH, Müller N, Schwartz DA, King TE, Lynch JP, Hegele R, Waldron J, Colby TV, Hogg JC (2003). Radiologic findings are strongly associated with a pathologic diagnosis of usual interstitial pneumonia. Chest.

[CR16] Kelly CA, Saravanan V, Nisar M, Arthanari S, Woodhead FA, Price-Forbes AN, Dawson J, Sathi N, Ahmad Y, Koduri G, Young A, British Rheumatoid Interstitial Lung (BRILL) Network (2014). Rheumatoid arthritis-related interstitial lung disease: associations, prognostic factors and physiological and radiological characteristics—a large multicentre UK study. Rheumatology (Oxford).

[CR17] Kim EJ, Elicker BM, Maldonado F, Webb WR, Ryu JH, Van Uden JH, Lee JS, King TE, Collard HR (2010). Usual interstitial pneumonia in rheumatoid arthritis-associated interstitial lung disease. Eur Respir J.

[CR18] Morelli S, Barbieri C, Sgreccia A, Ferrante L, Pittoni V, Conti F, Gualdi G, Polettini E, Carlesimo OA, Calvieri S (1997). Relationship between cutaneous and pulmonary involvement in systemic sclerosis. J Rheumatol.

[CR19] Morgenthau AS, Padilla ML (2009). Spectrum of fibrosing diffuse parenchymal lung disease. Mt Sinai J Med.

[CR20] Nozu T, Kondo M, Suzuki K, Tamaoki J, Nagai A (2009). A comparison of the clinical features of ANCA-positive and ANCA-negative idiopathic pulmonary fibrosis patients. Respiration.

[CR21] Olson AL, Swigris JJ, Sprunger DB, Fischer A, Fernandez-Perez ER, Solomon J, Murphy J, Cohen M, Raghu G, Brown KK (2011). Rheumatoid arthritis-interstitial lung disease-associated mortality. Am J Respir Crit Care Med.

[CR22] Ostojić P, Damjanov N (2006). Different clinical features in patients with limited and diffuse cutaneous systemic sclerosis. Clin Rheumatol.

[CR23] Park JH, Kim DS, Park IN, Jang SJ, Kitaichi M, Nicholson AG, Colby TV (2007). Prognosis of fibrotic interstitial pneumonia: idiopathic versus collagen vascular disease-related subtypes. Am J Respir Crit Care Med.

[CR24] Rubio-Rivas M, Royo C, Simeón CP, Corbella X, Fonollosa V (2014). Mortality and survival in systemic sclerosis: Systematic review and meta-analysis. Semin Arthritis Rheum.

[CR25] Szücs G, Szekanecz Z, Zilahi E, Kapitány A, Baráth S, Szamosi S, Végvári A, Szabó Z, Szántó S, Czirják L, György Kiss C (2007). Systemic sclerosis rheumatoid arthritis overlap syndrome: a unique combination of features suggests a distinct genetic, serological and clinical entity. Rheumatology (Oxford).

[CR26] Turesson C, O’Fallon WM, Crowson CS, Gabriel SE, Matteson EL (2003). Extra-articular disease manifestations in rheumatoid arthritis: incidence trends and risk factors over 46 years. Ann Rheum Dis.

[CR27] White B (2003). Interstitial lung disease in scleroderma. Rheum Dis Clin North Am.

